# Pre-trial qualitative work with health care professionals to refine the design and delivery of a randomised controlled trial on kidney care

**DOI:** 10.1186/s13063-019-3281-z

**Published:** 2019-04-16

**Authors:** Samantha Husbands, Fergus Caskey, Helen Winton, Andy Gibson, Jenny L. Donovan, Leila Rooshenas

**Affiliations:** 1 Population Health Sciences, Bristol Medical School, University of Bristol, 1-5 Whiteladies Road, Bristol, BS8 1NU UK; 2 Population Health Sciences, Bristol Medical School, University of Bristol, Canynge Hall, 39 Whatley Road, Bristol, BS8 2PS UK; 3 Department of Health and Applied Social Sciences, University of West of England, Bristol, UK

**Keywords:** RCT, RCT design, Pre-trial, Qualitative, Kidney Care, Health-care Professionals, Patient and Public Involvement

## Abstract

**Background:**

Recruitment to randomised controlled trials (RCTs) is challenging. Pre-trial qualitative research provides insights into the feasibility and acceptability of proposed trial designs and delivery; however, this is rarely conducted. This paper reports on work undertaken in advance of the *Prepare for Kidney Care* trial (formerly *PrepareME*), which compares preparing for dialysis with preparing for conservative care for patients with chronic kidney disease. The paper describes how the findings refined plans for the forthcoming trial.

**Methods:**

Semi-structured interviews were undertaken with health-care professionals involved in delivering or recruiting to the trial. Interview findings were considered in relation to observations of a patient advisory group workshop and introductory site visits, which were set up to present the trial to professionals involved in the internal pilot phase of the RCT. The use of findings and input from multiple sources was intended to support suggested refinements to the forthcoming trial. The findings were fed back to the trial management group and other expert stakeholders.

**Results:**

Sixteen health-care professionals were interviewed, and one patient advisory group workshop and six introductory visits to sites involved in the internal pilot were observed. The professionals interviewed included renal consultants, nurses and renal social workers. Key themes identified from the interviews, supported by the observations, were concerns around the eligibility criteria, the feasibility of the trial intervention, imbalances in the presentation of the trial arms, and anticipated recruitment issues arising from patients’ and clinicians’ preferences for one arm or the other. Changes to the design were made in response, including to the content of the intervention, the presentation of the trial arms and the name of the RCT.

**Conclusions:**

This study highlights the value of carrying out pre-trial work with health-care professionals to identify issues with delivering the proposed trial. This work can be particularly valuable in trials of new interventions, for which the barriers to their integration into routine care are unknown. This work has important implications for facilitating the identification of further obstacles in the main RCT. We suggest that pre-trial qualitative work is undertaken to address design issues early on, in addition to ongoing qualitative research to monitor the emergence of obstacles affecting recruitment.

## Background

Recruitment to randomised controlled trials (RCTs) is challenging, with only 56% of publicly funded RCTs in the UK reaching recruitment targets [1]. The reasons for recruitment failure often relate to issues around trial design and delivery, including logistical problems and equipoise issues relating to the trial arms [2, 3]. Briel and colleagues suggested that 89% of obstacles leading to the discontinuation of RCTs could be avoided if issues were identified and addressed during the trial planning stages [3]. Patient and public involvement (PPI) at the design stage has been reported as having a positive impact on RCT recruitment through, for example, suggesting ways to make the trial more attractive to potential participants [4]. Several studies have also highlighted the benefits of undertaking qualitative research at the pre-trial phase, with a view to refining study design and exploring the acceptability and feasibility of carrying out proposed trial processes [5, 6]. O’Cathain and colleagues [5] noted, however, that very little of this type of pre-trial research is undertaken, despite its potential to optimise trial design and recruitment. This particularly applies to research with health-care staff involved in delivery and recruitment to RCTs [7].

This article reports on pre-trial qualitative work carried out in advance of recruitment to the *Prepare for Kidney Care* RCT. Originally, and at the time of undertaking this qualitative research, the RCT was named *PrepareME*, and was set up in response to uncertainty around the survival and health-related quality of life benefits associated with different approaches to managing end-stage chronic kidney disease in patients aged 80 years and over, and those aged 65 years and over with other comorbidities [8]. A preceding UK-based mixed-methods study showed great variability in how these patients were being treated in National Health Service (NHS) hospitals around the country, in terms of whether they prepared for (and started) dialysis or conservative care [9]. Given these uncertainties, the trial aimed to investigate the relative clinical and cost-effectiveness of preparing patients for dialysis versus preparing them for conservative care [8].

The aim of this qualitative pre-trial work was to explore the acceptability and feasibility of conducting the *PrepareME* trial from the perspective of the clinical professionals who would be delivering it. This was with a view to refining the presentation of the RCT and the protocol to optimise recruitment and retention, and particularly, to identify barriers to integrating the trial into routine care.

### Trial context

Patients eligible for inclusion in *PrepareME* needed to have existing or newly diagnosed stage 5 chronic kidney disease (CKD), with an estimated glomerular filtration rate (eGFR) (i.e. kidney function) of <15 mL per min per 1. 73 m^2^. Patients also needed to be either 80 years of age or over, or 65 years of age and over with other health comorbidities (Table 1).

**Table 1:** *PrepareME* eligibility criteria: inclusion criteria

*PrepareME* patient eligibility criteria	
Patients known to renal services with new or existing stage 5 CKD (eGFR <15, with at least one result confirming this in the last 12 months) and - Aged 65+ with a World Health Organisation (WHO) performance status 3+, or - Aged 65+ with a Davies co-morbidity score 2+, or - Aged 80+.	
WHO performance status classification: 0 = Fully active, able to carry out all normal activities without restriction; 1 = Restricted in physically strenuous activity but ambulatory and able to carry out work of a light or sedentary nature; 2 = Ambulatory and capable of all self-care but unable to carry out any work activities; up and about more than 50% of waking hours; 3 = Symptomatic and in a chair or in bed for greater than 50% of the day but not bedridden; 4 = Completely disabled; cannot carry out any self-care; totally confined to bed or chair. Davies co-morbidity score: Each of the following scores one point: Malignancy, ischaemic heart disease, peripheral vascular disease (including stroke), left ventricular dysfunction, diabetes mellitus, systemic collagen vascular disease, other significant pathology (including COPD, cirrhosis, psychiatric illness or HIV).	

*CKD* chronic kidney disease, *COPD* chronic obstructive pulmonary disease, *eGFR* estimated glomerular filtration rate, *HIV* human immunodeficiency virus, *WHO world health organisation*

**Fig. 1:**
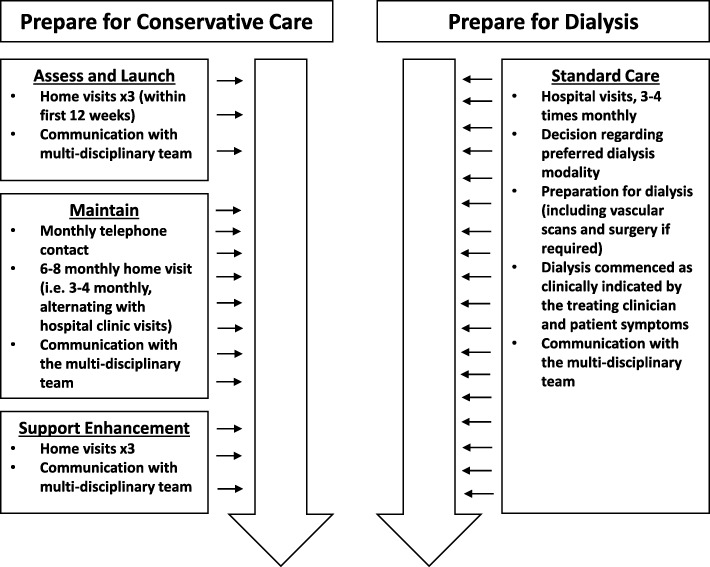
The *PrepareME* trial pathways

The original trial arms compared in the *PrepareME* RCT were *Prepare for dialysis* and *Prepare for conservative care* (Fig. 1). The *Prepare for dialysis* arm was described as standard care, referring to usual practice in the recruiting site (most likely including surgery for dialysis access, decisions regarding patients’ preferred dialysis modality and regular hospital clinic visits). *Prepare for conservative care* was a newly proposed treatment pathway, developed specifically for the trial because previous research had found variation in the content and delivery of conservative care pathways across UK NHS renal units [9]. The intervention consisted of three phases:

- Assess and launch entailed evaluating patients’ needs through advanced CKD assessments and developing a plan of action to meet these.
- Maintain involved implementing the plan of action whilst continuing to review and respond to symptoms.
- Support enhancement focused on delivering end-of-life care to patients, including relieving pain and symptoms and advanced-care planning.

A key element of *Prepare for conservative care* was the delivery of care within patients’ homes, aimed at supporting patients whilst reducing the number of hospital visits. Sites were required to offer up to three home visits to patients within the first 12 weeks of allocation as part of the assess and launch phase. The maintain phase consisted of intermittent home and hospital clinic visits, with monthly telephone contact from a health-care professional. The support enhancement phase was designed to include up to three home visits (Fig. 1).

**Fig. 2:**
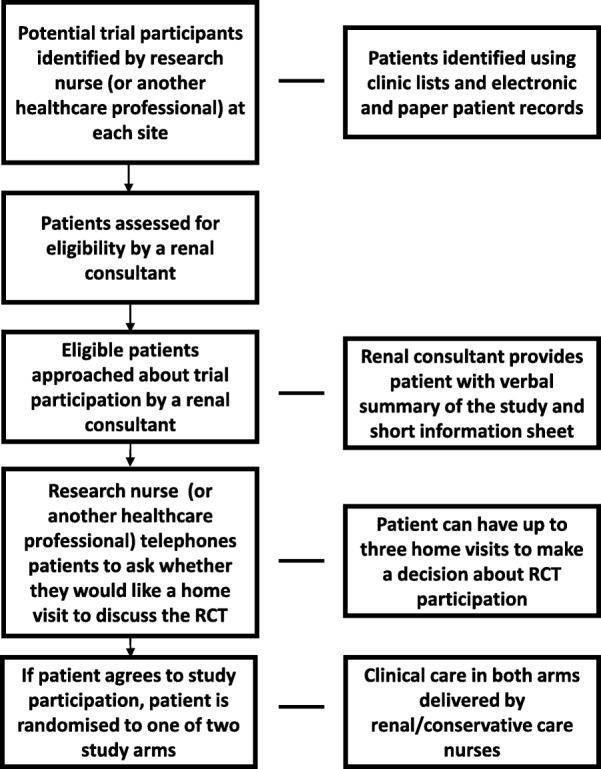
The *PrepareME* patient recruitment pathway. RCT randomised controlled trial

Recruitment was designed to be led by research nurses, but multiple health-care professionals could be involved, including clinical care nurses, renal consultants and renal social workers. A summary of the intended recruitment pathway—from identification of patients to obtaining written consent—is shown in Fig. 2. A research nurse (or another health-care professional) was required to identify potentially eligible trial participants, with eligibility being confirmed by a renal consultant. Renal consultants would initially approach eligible patients about trial participation, by providing a brief verbal summary of the study, and a short invitation letter and introductory patient information sheet. A research nurse (or other health-care professional) then contacted the patient by telephone to see if they were interested in receiving a home visit to discuss the study. Up to three home visits to provide information about the RCT were permitted. Conducting these recruitment discussions at home, rather than in clinic, was a suggestion made by PPI co-applicants prior to the trial, who felt that this would make it easier for friends, family and carers to be involved in making the decision about trial participation.

An internal pilot phase was built into the trial for the first year of recruitment, which involved the RCT being implemented in a subset of centres, to pilot the logistics and identify barriers to delivering the trial prior to the main study [10]. A follow-up cohort study using the UK renal registry was also set up alongside the RCT to capture observational follow-up outcome data for patients who declined to participate in the RCT.

## Methods

### Study design

This was a qualitative research study, consisting of semi-structured interviews with health-care professionals. The qualitative research also drew upon observations of the *PrepareME* patient advisory group (PAG) workshop and introductory site visits. Ethical approval for the qualitative study was obtained from the research ethics committee of the Faculty of Health Sciences at the University of Bristol (reference 44001).

### Semi-structured interviews

#### Sampling and recruitment

A purposive sampling strategy was adopted to target professionals who were delivering existing renal care and treatment to the eligible patient population, and those who were anticipated to influence the recruitment and retention of patients (i.e. assessing patients for eligibility, introducing trial participation to patients and delivering patient care as required by the RCT arms). Sampling aimed to achieve wide variation by capturing individuals in different clinical roles across all six sites involved in the internal pilot phase of the RCT [11, 12]. Sites were spread across various regions in England and were selected for the internal pilot phase because the trial team knew that they were offering different models of conservative care to patients. Participants for the interviews were identified through introductory site visits and existing links with the chief investigator (CI) and the trial management group (TMG). Snowball sampling was also employed, in which participants were asked to suggest other individuals who may have a role in delivering the trial in their centres [13].

Interviews were undertaken in waves to allow subsequent sampling to be guided by previous data collection and with the intention to develop themes [14]. Staff were invited to take part in an interview via email and were sent a study information sheet. Those who agreed to take part were asked to suggest a convenient date and time for the interview. Attempts were made to organise the interviews after staff had received their introductory site visit and had had the study explained to them by the CI, to allow them time to absorb and understand the protocol. Those who did not respond were sent a reminder by email 2 weeks after the initial contact.

### Data collection

Semi-structured interviews were conducted by SH between February 2017 and March 2017, either face to face or over the telephone. SH is a qualitative researcher by background, with a PhD in social sciences. She had no prior relationship with the health-care staff approached for interview and was not involved in the early development of the trial design. A flexible topic guide was developed from previous studies. For instance, questions relating to equipoise and the trial eligibility criteria were informed by the previous integration of the Quintet Recruitment Intervention (QRI) into trials, since these issues have been commonly explored in the early stages of RCTs [15, 16]. Other questions covered details about how patients are managed in routine clinical practice, participants’ perceived acceptability and the relevance of the study design and intervention, and views on the feasibility of trial conduct (especially recruitment). The topic guide was also reviewed with other researchers who had prior experience of conducting research with renal professionals. We did not pilot the topic guide, but we added further prompts, probes and questions as data collection proceeded.

Interviews continued until data saturation occurred, determined to be the point at which no new issues were being introduced by new participants, and the views within the key themes identified (i.e. those with implications for trial design and delivery, as reported in this paper) were being replicated across different health-care professionals from different backgrounds and contexts [17]. Interviews were audio-recorded after the participant had provided written informed consent and they were transcribed verbatim. The mean length of an interview was 44 min (ranging from 27 to 69 min).

### Data analysis

Transcripts were checked against audio-recordings for accuracy and imported into NVivo 10 for coding. Interviews were analysed using methods of constant comparison, which require new data to be continually compared with existing data to enhance understanding and explore relationships between themes [14]. Interview data were coded line by line. Themes were initiated and evolved through the continual comparison of data. Interviews were undertaken in batches of two or three so that existing findings could inform subsequent data collection, and the coding structure could be updated to encompass new themes. A descriptive account was generated to summarise themes and set out similarities and differences between participants’ perspectives. Analysis was undertaken by two experienced qualitative researchers, primarily by SH, with 10% of transcripts coded independently by LR to promote reliability [18]. SH and LR met regularly to discuss the findings and to ensure that they agreed on the overarching issues emerging from the interviews [19]. Conversations focused on whether adaptations should be made to the overall coding strategy to ensure that no important themes were being missed. Findings from the interviews were summarised in a report for the CI and then presented to broader trial stakeholders at an expert consensus meeting, with the aim of finalising trial details and refining the study protocol. This meeting took place on 17 March 2017 and was attended by individuals involved in study oversight (TMG members), researchers working on the project, study co-applicants and PPI representatives.

### Observations of introductory site visits and the PAG workshop

Observations of introductory site visits and the PAG workshop were undertaken to capture the views of additional key stakeholders on the trial design. Key issues identified from observations with further health-care professionals and patient representatives helped to inform changes to the interview topic guide, which could be further explored in subsequent interviews. This was intended to triangulate different sources of information [20] and to explore whether consistent views were emerging from different stakeholders and forums (i.e. group versus individual insights). Considering information from these different sources helps to confirm emerging insights from any one source (i.e. strengthening credibility) and build a more comprehensive understanding of the possible barriers and facilitators to the successful delivery of the trial. This was important for presenting a confident rationale for any proposed changes to the trial design or protocol. In some cases, findings from the qualitative interviews were fed into the observed meetings (i.e. the PAG) for discussion.

### Introductory site visits

The CI, trial manager and qualitative researcher(s) conducted introductory site visits at all six renal units involved in the internal pilot phase of the RCT. The visits took place prior to the RCT, between 9 January and 3 April 2017. The purpose of these visits was to introduce the details of the RCT, explore how the trial processes might work in practice and address any questions or queries. These visits were attended by clinical and recruitment staff, including site principal investigators, research nurses, research co-ordinators, and dialysis and conservative care nurses.

### Patient advisory group workshop

A face-to-face *PrepareME* PAG consisting of PPI representatives was set up in advance of the trial’s commencement to obtain feedback on the clarity and presentation of patient information sheets and questionnaires. Members of the PAG were given a summary of the proposed RCT at the beginning of the workshop. The workshop was chaired by the CI on 7 March 2017 (i.e. after most of the interviews had been conducted) and lasted approximately 2 hours.

### Output from the observations

**Fig. 3:**
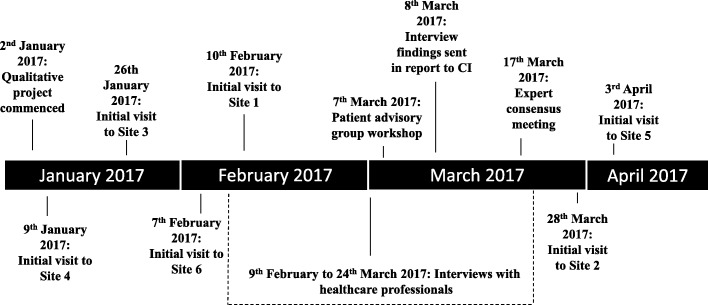
Timeline of qualitative data collection and feedback

SH and LR took detailed notes at both the introductory site visits and the PAG meeting. During the site visits, general points relating to attendees’ reactions to aspects of the study and any questions or queries that arose were noted. In the PAG, general notes about the issues raised during the discussions were recorded and official written minutes were taken by the trial manager. Notes and minutes from site visits and the PAG workshop were collected, and the key issues identified were compared to those from the interview data, drawing on techniques of constant comparison (as outlined above) [14]. A timeline of data collection and written and oral presentations of the qualitative research findings is presented in Fig. 3.

## Results

### Participants

Sixteen health-care professionals were approached for the semi-structured interviews, and all agreed to take part. Interviews involved participants from four of the six acute NHS trusts participating in the internal pilot phase of *PrepareME*. Those interviewed included renal consultants (*n* = 6), CKD nurses (*n* = 4), conservative care nurses (*n* = 2), palliative care nurses (*n* = 2) and renal social workers/counsellors (*n* = 2). Three of the renal consultants were the principal investigators for the trial, and two of these were also co-applicants. Interview participant characteristics, including their anticipated roles in the RCT, are shown in Table 2. The PAG workshop was attended by eight PPI representatives, two PPI specialists, the CI and four other members of the TMG.

**Table 2:** Interview participants’ professional roles and anticipated role in RCT

Professional roles	Participant IDs	Sites	Anticipated role in the trial
Conservative care nurses	P1, P3	4	Delivering *Prepare for conservative care*
Palliative care nurses	P2, P9	3	Delivering *Prepare for conservative care*
Renal consultants	P4, P5, P7, P8, P10, P16	3, 4, 6	Identifying patients for the RCT (*n* = 6) Trial principal investigator and trial co-applicant (*n* = 2), co-applicant (*n* = 2) or trial principal investigator only (*n* = 1)
Chronic kidney care nurses	P6, P12, P14	1, 3	Delivering *Prepare for dialysis*
Renal education nurse	P11	4	Introducing treatment pathways to patients (prior to the RCT)
Renal social worker/counsellor	P13, P15	6	Delivering care or support within *Prepare for conservative care*

*RCT* randomised controlled trial

Key themes identified from the interviews are presented below with illustrative quotes throughout. Identifiers (P1–P16) have been used to protect participant and recruitment site identities. Themes that were also supported by discussions arising in the PAG and introductory site visits are indicated. The final part of this section discusses changes made to the trial design because of the research.

### Concerns around the trial eligibility criteria

#### Clinical criteria

Some participants expressed concerns about the proposed trial inclusion criteria (Table 1), specifically the need for patients to have an eGFR of <15. These concerns were discussed by consultants and nurses across two of the four recruiting sites and flagged in introductory visits to these sites (sites 3 and 4). In general, concerns aligned with the idea that patients have typically already made decisions regarding their preferred treatment pathway by the time their kidney function had declined to this level:

P8: “*Less than 15, an eGFR, is quite late. … In reality, a good few of our patients … would have made their decision before their eGFR is less than 15, and we would have been encouraging that.”*

#### Age and health status

Professionals questioned the appropriateness of the age and health-related criteria, suggesting that particular groups of eligible patients might be more suited to one treatment pathway over the other, although views were not shared in relation to sub-groups of eligible patients. For example, a quarter of those interviewed (all conservative care nurses or renal social workers) stated that patients aged 80 and over would be more suitable for or more likely to opt for conservative care (P9), whilst another quarter of those interviewed (mostly consultants) suggested it was favourable for patients aged 80 and over to receive dialysis if they were deemed to be otherwise healthy (P8):

P9 (palliative care nurse): *“Those 80 or over, the evidence has shown there is very little benefit of having dialysis and it actually can impact their quality of life.”*

P8 (renal consultant): “S*he’s 85, plays golf twice a week. … She’s absolutely fit as a flea. … I might be tempted to weigh my judgement towards dialysis, because she has such a good quality of life.”*

Concerns were also expressed in relation to patients aged 65 and over with comorbidities. Just under half of interview participants conveyed discomfort with including these patients in the trial, as they were deemed to be too young to receive conservative care (irrespective of their co-morbidities):

P6 (CKD nurse): “*It’s a fairly young cut-off. … I think there are probably many people that are [in the] 65 age group would recommend the first stage, trying dialysis first.”*

However, a few participants, particularly nurses who delivered conservative care, were of the view that patients aged 65 with severe comorbidities were more suited to conservative care:

P3 (conservative care nurse): “*65 and then your comorbidity’s two plus. … I’d probably say they’re the ones who don’t do so well on dialysis. So, maybe in an ideal world … they probably would be better for opting for conservative [care].”*

The issues around eligibility emerging from the interviews tended not to be raised in the introductory site visits.

### The trial intervention: *Prepare for conservative care*

#### Intervention content

Comments on the intervention focused on the delivery of the home visits, with most participants supportive of their value and suggesting that it was useful to evaluate patients’ care needs within their own home. Some believed home visits allowed patients ownership and control:

P1 (conservative care nurse): *“You can make an assessment … how they’re managing on a day-to-day basis. … Do you need district nurses or a package of care coming in?”*

P9 (palliative care nurse): *“People are often more comfortable talking in their own home. They have a bit more control.”*

Most discussion around the home visits focused on the cost of delivery, as home visits were considered as excess treatment costs and therefore, were not covered by the trial. Professionals commented on the ability of sites to deliver home visits, with issues raised relating to the time and money required for staff to undertake additional visits (over and above those offered in routine practice), both in terms of how the visits would be funded, and issues around the availability of staff to deliver these:

P6 (CKD nurse): *“We will need to work out how we can offer [home visits]. I think everybody’s in the same situation. ... We’re really stretched in terms of resources.”*

However, participants from two of the sites (sites 4 and 6), which were delivering home visits regularly as part of their standard care, appeared more optimistic about their ability to deliver the intervention:

P15 (renal social worker): *“I don’t think [the home visits] would be a problem. These are patients that are our patients anyway, so we would be seeing them … because we do that work anyway.”*

Despite general support for integrating home visits into the *Prepare for conservative care* pathway, half of those interviewed expressed that the number of specified home visits (three) was too intensive, suggesting that this set-up might not suit the wishes and lifestyle of all patients:

P2 (palliative care nurse): *“It may be too much for some. … You wouldn’t go three times into the home of someone who didn’t want you in their house and didn’t need you.”*

Hesitations about the volume or frequency of home visits tended not to be expressed at the introductory site visits (with one exception, site 5, which suggested that the number of visits might be problematic in terms of the resources they had available).

#### Intervention name

Around a third of participants suggested that the name *Prepare for conservative care* implied a passive approach to delivering care, in contrast to active implications of *Prepare for dialysis*. As such, participants suggested alternative names that they felt might mitigate the possibility of patients misinterpreting what conservative care entailed:

P6 (CKD nurse): “*We quite like having ‘maximum’ [conservative management] because it’s … having a proactive approach to it, isn’t it? … I think there is, maybe, an element of just emphasising that we are still doing something although it’s conservative. … I think … just ‘conservative’ is maybe a bit lacking.”*

Observations from the PAG workshop supported this finding after the CI (chair) asked PPI representatives for their views on the name of the intervention. This was not on the original meeting agenda, but the qualitative team had informally fed back some of the interview findings to the CI. Several of the attendees suggested that the term ‘conservative care’ held negative connotations, with implications that patients would be left to die on this pathway. PPI representatives similarly preferred the proposed use of more active terms, including the suggested ‘supportive care’ and ‘active clinical care’.

#### Balancing the trial pathways

Interview participants commented on the presentation of the study pathways within the RCT patient information sheet, particularly the differences in the phases of care represented in the study design (Table 1). An interview participant (P2) suggested that the lack of portrayal of a palliative care element within the *Prepare for dialysis* arm gave the impression that a patient’s health would not decline if they were randomised to this pathway:

P2 (palliative care nurse): *“Have we got any advanced care planning built into the preparation for dialysis? I think it would be really helpful to know what happens if you were recognised as dying in both arms.”*

Comments from the PAG supported this, as members observed that only the *Prepare for conservative care* pathway appeared to lead to death (as stated in the official PAG minutes).

### Issues with recruitment to *PrepareME*

Participants were asked for their views on recruitment to *PrepareME*, particularly perceptions of recruitment difficulties. Several potential threats to recruitment arose, some specifically in the context of compromising recruitment, whilst others were mentioned without reference to recruitment, but could be seen to complicate or threaten trial participation. A few participants commented directly on the name of the trial, with a consultant (P7) suggesting that it might have negative connotations for other clinical staff:

P7 (renal consultant): *“I’ve had some person refer to it as the ‘prepare me to die’ study. Seriously.”*

A further recruitment barrier identified directly by those interviewed were patient preferences, with around half of participants suggesting that eligible patients would have strong views about their preferred treatment pathway, and thus may not agree to randomisation:

P8 (renal consultant): “*I’m always surprised how fixed people’s opinions are, and when you do try to explore them, yes, occasionally you get people to open up their minds. … But sometimes, it’s almost as if like, they’re not really listening.”*

However, a few professionals stated views to the contrary, believing that most patients do not have fixed or well-informed views:

P16 (renal consultant): “*People have read about dialysis and have decided it’s not for them. But it’s not always an informed decision. So, even if they say they don’t want to have dialysis … we wouldn’t necessarily take that at face value because, in my experience, a lot of people … change their minds.”*

Around a quarter of those interviewed suggested clinician equipoise would be an issue for other clinicians involved in the study, implying that when initially discussing the trial with potential participants, clinicians may unintentionally introduce treatment pathways in a biased way because of their belief that patients might be more suited to one intervention over the other:

P15 (renal social worker): “*[Clinicians] provide unclear or skewed information to try to encourage someone to go down the conservative care pathway—not for any underhand reason but because they do sincerely believe that for that individual that would be the right route. … That happens in practice everywhere.”*

Potential issues around equipoise were also apparent in some participants’ suggestions that they would be uncomfortable randomising patients. Some of these accounts were linked to comments on the registry follow-up study. Without prompting, a quarter of participants discussed the value of the registry study, but some also gave the impression that the registry could be a good substitute for the RCT:

P5 (renal consultant): *“I think that, actually, [the registry study is] a saviour for me. … If [patients] don’t want to be randomised they could be followed up.”*

### Changes to the trial design

Findings from the semi-structured interviews were analysed and summarised in a report for the CI and later discussed with trial stakeholders at an expert consensus meeting (see Fig. 3 for a timeline of events). At this meeting, the qualitative team presented the key issues raised by the health-care professionals, using anonymised quotations from the interviews as evidence. Experts were then given the opportunity to give their thoughts on these issues, which were recorded in the official meeting minutes. This included a consensus for changing the name of the RCT and the name of the *Prepare for conservative care* arm. In a series of further meetings between the CI, TMG and qualitative team, the feedback from the expert consensus meeting and the insights from the introductory site visits and the PAG workshop were considered and used in support of making practical changes to the trial design and presentation, and amendments to the study protocol. These changes are discussed below.

### Changes to the trial arms

#### The intervention content

Concerns about the practicalities and resource implications of delivering the home visits were considered and discussed in depth, with the CI taking an active role in negotiating local solutions that worked for staff in each site—a time-consuming but important process that was not anticipated at the funding acquisition stage. In response to these issues, and concerns about patients’ potential reluctance to accept frequent home visits, the CI proposed to amend the protocol by reducing the number of compulsory home visits in the earliest assess phase of the pathway. The number of visits was changed from three to a minimum of one, allowing the final number to be influenced by the individual requirements and preferences of each patient.

#### The intervention name

The CI and TMG decided to reconsider the name of the intervention, taking on board interview participants’ and the PAG members’ suggestions for clarifying the arm’s active nature of delivering care. ‘Prepare for responsive management’ was put forward by the qualitative team, following discussions with the CI about what activities the intervention arm entailed. The term ‘responsive’ was proposed to communicate the responsive nature of monitoring and reacting to patients’ symptoms in this care pathway. The term ‘management’ was intended to reflect the active component of the intervention, which interview participants and the PAG felt was previously absent. The proposed revised name was approved by the CI, TMG and PPI representatives.

#### Presentation of the trial arms

**Fig. 4:**
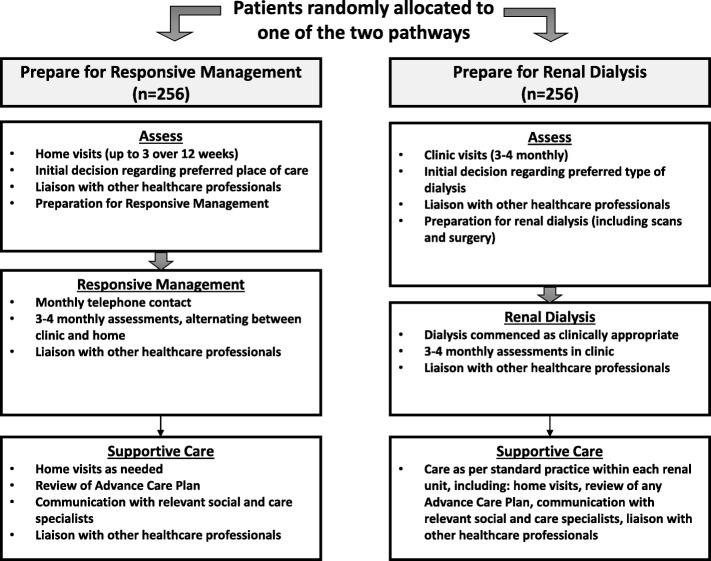
*Prepare for Kidney Care*: revised trial pathways

**Fig. 5:**
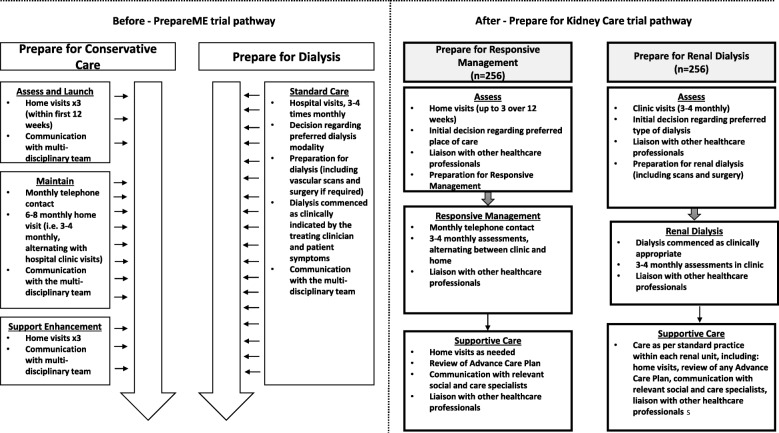
Comparison of trial pathways

Perceived imbalances between the trial arms were addressed through changes to how the *Prepare for dialysis* pathway was presented. The CI and qualitative research team worked together to redesign the trial arms, to illustrate the balance between the arms and ensure the information presented accurately reflected the CI and TMG’s plans for what the trial arms would involve. These changes constituted adding an assess phase (formerly absent), and a supportive care phase (formerly, support enhancement) to the *Prepare for dialysis* arm, to mirror the phases presented in *Prepare for conservative care*. Detailed descriptions of activities in each of the phases used the same terminology for each of the trial arms as far as possible, with a view to making the similarities and key differences between the trial arms clear. Figure 4 shows the revised presentation of the trial arms, and the trial pathways before and after the qualitative work are given in Fig. 5.

### Name of the RCT

The CI and TMG felt the name of the RCT should be revised, in response to some interviewees’ perceptions that *PrepareMe* had possible negative connotations. A change to the trial name was also supported by the research ethics committee (March 2017). Potential alternatives were devised and circulated to the PAG for their feedback. ‘Prepare for kidney care’ was the option favoured by most PPI representatives and the trial team.

## Discussion

This qualitative pre-trial study was designed to explore health-care professionals’ perspectives on the design, relevance and feasibility of delivering and recruiting to the *PrepareME* RCT. The findings, supported by observations of a PAG workshop and introductory site visits, informed changes to the trial design and protocol.

Findings from the interviews on trial design focused on logistical issues, particularly concerns about the ability to deliver home visits as part of the intervention. Professionals also discussed issues with the trial inclusion criteria, namely approaching patients for study participation when their eGFR < 15, as some commented that patients would have already made treatment decisions in routine care. The presentation of the RCT and the trial arms, specifically the name of the trial (*PrepareME*) and the intervention (*Prepare for conservative care*), were suggested to have negative connotations, which could impact patients’ interpretations and willingness to participate. Further issues concerned perceived imbalances in the presentation of the trial pathways, as interview participants and PAG members highlighted that patients’ health appeared to decline only in the *Prepare for conservative care* pathway (rather than in both pathways). Factors that were anticipated to hinder recruitment included expectations that patients would have treatment preferences and concerns about clinical professionals’ perceptions of equipoise. Equipoise issues were indirectly implied through professionals’ comments on the registry follow-up study, with some staff suggesting that this non-randomised study could provide a more comfortable substitute for the RCT.

As an outcome of this research, organisational and design issues were mostly addressed at the trial outset, for example, updating the protocol to add flexibility to home visits. Changes were made to the name of the trial and the conservative care pathway (renamed *Prepare for Kidney Care* and *Prepare for responsive management*, respectively), and the presentation of the trial arms was refined to communicate clearly the constituents of each arm and to convey equipoise. Other issues were more difficult to address immediately, particularly anticipated recruitment problems, as it was unclear whether these issues would occur, and some of these issues were sensitive and complex, apparently related to the personal views of health-care professionals towards the RCT. These have been recognised elsewhere as the emotional and intellectual aspects of trial recruitment [21–24], including recruiter struggles with equipoise [21–23] and reluctance to explore patient preferences [22].

A QRI was later integrated into trial recruitment within *Prepare for Kidney Care*, with the aim to understand recruitment in depth, as it proceeds, and to identify factors that are hindering trial uptake [16]. The findings are used to inform strategies to optimise recruitment, often comprising support and training of recruiters [25]. Recruitment and the QRI are now underway for *Prepare for Kidney Care*, and whilst the QRI aims to identify new recruitment obstacles, issues highlighted in this pre-trial work have informed some of the focus of investigation, allowing the team to monitor these issues and act rapidly if they arise.

The key focus of this study was on the views of health-care professionals on the proposed RCT. This was a strength, as staff were able to offer real insights into problems with integrating the trial into routine practice. As with many RCTs, patient involvement was—and continues to be—essential in the *Prepare for Kidney Care* study; however, the contributions from staff delivering existing care included unique (insider) insights into key organisational issues likely to affect the proposed trial’s execution. A review of pre-trial work found that most existing qualitative studies on the acceptability of trial interventions were from the perspective of patients, with few including professionals [7]. We were able to identify only one other pre-trial study, the Bluebelle Study [6], that used interviews with health-care professionals to assess the feasibility of trial design and recruitment. The interviews sought feedback on a proposed trial to compare types of surgical wound dressings, with findings indicating that a proposed comparator was not routinely used in practice, resulting in a change to the trial arms.

Other studies have instead integrated qualitative methods during recruitment (i.e. the QRI), with most of these emphasising problems with recruitment practices rather than design, including logistical issues (e.g. screening, assessing eligibility and approaching patients) and the communication of trial information [26–32]. Design issues were identified within only one study (ProtecT—Prostate testing for cancer and treatment) [26], specifically problems with the non-radical comparator arm. Like our work, the name of the arm was changed from ‘watchful waiting’ to ‘active monitoring’ to avoid non-interventionist connotations, and monitoring visits and tests were added into the active monitoring arm to balance the intensity of the intervention against its comparators. However, the drawback of exploring the acceptability of RCT design after recruitment is underway is that changes cannot always be made in time to have a positive impact on recruitment. This was demonstrated in a study by Ziebland et al. [33], who identified that the name of a trial (Spine Stabilisation Trial) did not suggest equivalence between its comparators by emphasising the surgical over the rehabilitation arm. The authors suggested that this contributed to recruiter misunderstandings of the trial design (i.e. not understanding that the treatments were equivalent), potentially negatively impacting recruitment discussions and uptake, and after the trial, affecting clinicians’ ability to interpret the trial’s results and apply them in practice.

There were several advantages to undertaking this pre-trial qualitative work in our study. We could address issues likely to affect recruitment and trial delivery in advance, which helped to maximise the research value, as the findings impacted specific trial delivery rather than generating future lessons [7]. The work supports the importance of an early investigation to ensure that sites have the appropriate infrastructure and resources to support trial delivery and procedures, to mitigate problems with trial conduct and to avoid delays [34]. We identified unanticipated resource constraints related to delivering the home visits in the intervention, suggesting qualitative pre-trial work to be particularly important when exploring potential practical and resource-related obstacles in trials aiming to deliver new interventions in existing NHS systems. This pre-trial work also allowed us to strengthen the relevance and acceptability of a new intervention for comparison (*Prepare for responsive management*) by drawing on the views of key staff and patient stakeholders [7]. Finally, undertaking early qualitative work can have the added advantage of encouraging improved collaborations between qualitative researchers and trial teams, facilitating ongoing work during trial recruitment, and potentially enhancing the integration and impact of qualitative research findings on trial conduct [35, 36].

This study has several limitations. We were able to conduct interviews with health-care professionals in only four of the six sites. This was due to delays in receiving local trust approval to begin recruitment at the remaining sites, which delayed introductory site visits and the opportunity for staff to hear about the study before being approached for interview. We allowed 6 months for data collection and analysis prior to recruitment commencing. However, we found that the timeline for completing the interviews was tight due to delays involved in gaining site approvals, the difficulties in obtaining health-care professionals’ time to conduct the interviews, and the time needed to feed back and implement the findings prior to recruitment. This meant, for example, that the findings had to be presented at the expert consensus meeting before all the interviews were complete, and not all interview findings could be fed into the PAG workshop for their reflections. Although the order in which we could undertake the interviews, observations and feedback session was restricted by the availability of the relative participants and stakeholders, we did ensure that we had complete input from all these groups before we made changes to the trial design.

Future studies would benefit from ensuring during the planning stages that they allow enough time and resources to organise stakeholder input, complete data collection and implement changes to the trial design prior to recruitment, including a draft timeline of when it would be optimal to conduct and present various aspects of the research with and to the various stakeholder groups. This may be facilitated by including the observation of PAG and introductory site meetings as formal data collection opportunities, allowing time for formal analyses and the chance also to present data from these investigations to stakeholders at the expert consensus meeting.

We did not interview patients to gather their views on the trial design prior to recruitment, which would have added empirical evidence of another key stakeholder group’s perspective. However, the rationale for interviewing health-care professionals first and foremost was to identify barriers to integrating the RCT into routine care, which might have prevented the trial running in participating sites. The views of patient representatives on trial design were gained through pre-trial workshops, and insights from the PAG workshop reported in this paper were considered in relation to the views of health-care professionals, which helped to support changes to the trial design. As part of the *Prepare for Kidney Care* QRI, interviews are now also underway with patients who have been approached for participation, for their reflections on the trial and recruitment processes. These interviews aim to explore the reasons behind patients’ participation decisions, highlighting any further design or recruitment obstacles. Had these patient interviews taken place at the pre-trial phase, they would have relied on patients considering participation hypothetically, which has limitations. We also did pre-trial work with only the first wave of sites involved in the pilot RCT. However, the challenges that have emerged from this pre-trial stage have informed the feasibility questionnaires and discussion points at new site initiation visits, which are helping the trial team to tease out and explore solutions to problems with trial conduct in advance.

A final limitation of this work is the inability to evaluate formally the influence of early changes made to the trial design on RCT delivery and recruitment. This would have been interesting from a trial methodology perspective. However, it is unlikely that the changes would have resulted in a negative impact, as changes focused on adding balance and flexibility to the design and delivery of the study arms and were grounded in data and insights emerging from the concerns of a range of health-care professionals and PPI representatives [7].

## Conclusions

This qualitative study has had important implications for the *Prepare for Kidney Care* RCT, identifying challenges related to its design and recruitment and allowing changes to be made to the RCT intervention and trial delivery in advance of the trial commencing. The benefits of carrying out pre-trial qualitative work is that it can inform specific changes to a trial design, potentially avoiding future issues with trial conduct, acceptability and recruitment. This study has also highlighted the value of conducting pre-trial work with health-care professionals who are delivering current clinical care, as they are able to offer key and knowledgeable insights into the potential barriers to integrating aspects of a trial into routine practice. Considering the views of health-care professionals in relation to other more typical non-empirical activities, such as introductory site visits and PPI activities, can provide an in-depth and comprehensive overview of issues that should (and can be) addressed in advance of trial delivery. We suggest that pre-trial qualitative work should be more often undertaken in addition to integrated qualitative research during recruitment to identify, monitor and address design and recruitment related issues in advance and as they arise.

## Abbreviations

CI: Chief investigator
CKD: Chronic kidney disease
COPD: Chronic obstructive pulmonary disease
eGFR: Estimated glomerular filtration rate
HIV: Human immunodeficiency virus
NHS: National Health Service
NIHR: National Institute for Health Research
PAG: Patient advisory group
PPI: Patient and public involvement
QRI: Quintet Recruitment Intervention
RCT: Randomised controlled trial
TMG: Trial management group
WHO: World Health Organisation
